# Swarm learning for decentralized artificial intelligence in cancer histopathology

**DOI:** 10.1038/s41591-022-01768-5

**Published:** 2022-04-25

**Authors:** Oliver Lester Saldanha, Philip Quirke, Nicholas P. West, Jacqueline A. James, Maurice B. Loughrey, Heike I. Grabsch, Manuel Salto-Tellez, Elizabeth Alwers, Didem Cifci, Narmin Ghaffari Laleh, Tobias Seibel, Richard Gray, Gordon G. A. Hutchins, Hermann Brenner, Marko van Treeck, Tanwei Yuan, Titus J. Brinker, Jenny Chang-Claude, Firas Khader, Andreas Schuppert, Tom Luedde, Christian Trautwein, Hannah Sophie Muti, Sebastian Foersch, Michael Hoffmeister, Daniel Truhn, Jakob Nikolas Kather

**Affiliations:** 1 Department of Medicine III, University Hospital RWTH Aachen, Aachen, Germany; 2 Pathology & Data Analytics, Leeds Institute of Medical Research at St James’s, University of Leeds, Leeds, UK; 3 Precision Medicine Centre of Excellence, Health Sciences Building, The Patrick G Johnston Centre for Cancer Research, Queen’s University Belfast, Belfast, UK; 4 Regional Molecular Diagnostic Service, Belfast Health and Social Care Trust, Belfast, UK; 5 The Patrick G Johnston Centre for Cancer Research, Queen’s University Belfast, Belfast, UK; 6 Department of Cellular Pathology, Belfast Health and Social Care Trust, Belfast, UK; 7 Centre for Public Health, Queen’s University Belfast, Belfast, UK; 8 Department of Pathology and GROW School for Oncology and Reproduction, Maastricht University Medical Center+, Maastricht, the Netherlands; 9 Division of Clinical Epidemiology and Aging Research, German Cancer Research Center (DKFZ), Heidelberg, Germany; 10 Clinical Trial Service Unit, Nuffield Department of Population Health, University of Oxford, Oxford, UK; 11 Division of Preventive Oncology, German Cancer Research Center (DKFZ) and National Center for Tumor Diseases (NCT), Heidelberg, Germany; 12 German Cancer Consortium (DKTK), German Cancer Research Center (DKFZ), Heidelberg, Germany; 13 Digital Biomarkers for Oncology Group (DBO), National Center for Tumor Diseases (NCT), German Cancer Research Center (DKFZ), Heidelberg, Germany; 14 Division of Cancer Epidemiology, German Cancer Research Center (DKFZ), Heidelberg, Germany; 15 Cancer Epidemiology Group, University Cancer Center Hamburg, University Medical Center Hamburg-Eppendorf, Hamburg, Germany; 16 Department of Diagnostic and Interventional Radiology, University Hospital RWTH Aachen, Aachen, Germany; 17 Institute for Computational Biomedicine, JRC for Computational Biomedicine, RWTH Aachen University, University Hospital Aachen, Aachen, Germany; 18 Department of Gastroenterology, Hepatology and Infectious Diseases, Medical Faculty of Heinrich Heine University Düsseldorf, University Hospital Düsseldorf, Düsseldorf, Germany; 19 Institute of Pathology, University Medical Center Mainz, Mainz, Germany; 20 Medical Oncology, National Center for Tumor Diseases (NCT), University Hospital Heidelberg, Heidelberg, Germany

**Keywords:** Predictive markers, Image processing, Machine learning, Biomedical engineering, Cancer imaging

## Abstract

Artificial intelligence (AI) can predict the presence of molecular alterations directly from routine histopathology slides. However, training robust AI systems requires large datasets for which data collection faces practical, ethical and legal obstacles. These obstacles could be overcome with swarm learning (SL), in which partners jointly train AI models while avoiding data transfer and monopolistic data governance. Here, we demonstrate the successful use of SL in large, multicentric datasets of gigapixel histopathology images from over 5,000 patients. We show that AI models trained using SL can predict *BRAF* mutational status and microsatellite instability directly from hematoxylin and eosin (H&E)-stained pathology slides of colorectal cancer. We trained AI models on three patient cohorts from Northern Ireland, Germany and the United States, and validated the prediction performance in two independent datasets from the United Kingdom. Our data show that SL-trained AI models outperform most locally trained models, and perform on par with models that are trained on the merged datasets. In addition, we show that SL-based AI models are data efficient. In the future, SL can be used to train distributed AI models for any histopathology image analysis task, eliminating the need for data transfer.

## Abstract (web summary)

A decentralized, privacy-preserving machine learning framework used to train a clinically relevant AI system identifies actionable molecular alterations in patients with colorectal cancer by use of routine histopathology slides collected in real-world settings.

## Main

AI is expected to have a profound effect on the practice of medicine in the next 10 years^1–4^. In particular, medical imaging is already being transformed by the application of AI solutions^5^. Such AI solutions can automate manual tasks in medical image analysis, but can also be used to extract information that is not visible to the human eye^6,7^. Digitized histopathology images contain a wealth of clinically relevant information that AI can extract^3^. For example, deep convolutional neural networks have been used to predict molecular alterations of cancer directly from routine pathology slides^8–13^. In 2018, a landmark study showed a first proof of principle for this technology in lung cancer^8^. Since then, dozens of studies have extended and validated these findings to colorectal cancer (CRC)^9,14,15^, gastric cancer^16^, bladder cancer^10^, breast cancer^13^ and other tumor types^10–12,17,18^. These methods expand the utility of H&E-stained tissue slides from routine tumor diagnosis and subtyping to a source for direct prediction of molecular alterations^3^.

AI models are data hungry. In histopathology, the performance of AI models increases with the size and diversity of the training set^16,19,20^. Training clinically useful AI models usually requires the sharing of patient-related data with a central repository^21,22^. In practice, such data sharing—especially across different countries—faces legal and logistical obstacles. Data sharing between institutions may require patients to forfeit their rights of data control. This problem has been tackled by (centralized) federated learning (FL)^23,24^, in which multiple AI models are trained independently on separate computers (peers). In FL, peers do not share any input data with each other, and only share the learned model weights. However, a central coordinator governs the learning progress based on all trained models, monopolizing control and commercial exploitation.

In the past 2 years, this limitation of FL has been addressed by a new group of decentralized learning technologies, including blockchain FL^25^ and SL^26^. In SL, AI models are trained locally, and models are combined centrally without requiring central coordination. By using blockchain-based coordination between peers, SL removes the centralization of FL and raises all contributors to the same level. In the context of healthcare data analysis, SL leads to equality in training multicentric AI models and creates strong incentives to collaborate without concentrating data or models in one place. This could potentially facilitate collaboration among several parties, hence generating more powerful and more reliable AI systems. Ultimately, SL could improve the quality, robustness and resilience of AI in healthcare. However, SL has not been systematically applied to medical image data in oncology. In particular, it has not been applied to histopathology images, a common data modality with a high information density^3^.

In this study, we examine whether SL can be used for AI-based prediction of molecular alterations directly from conventional histology images. To investigate this, we perform a retrospective multicentric study. As pathology services are currently undergoing a digital transformation, embedding AI methods into routine diagnostic workflows could ultimately enable the prescreening of patients, thereby reducing the number of costly genetic tests and increasing the speed at which results are available to clinicians^27^. The prediction performance of such systems increases markedly by training on thousands rather than hundreds of patients^19,20^. We hypothesize that SL could be a substitute for the centralized collection of data from large patient cohorts in histopathology, improving prediction performance^20^ and generalizability^22^ without centralizing control over the final model.

## Results

### SL can be used to train AI models for pathology

**Fig. 1:**
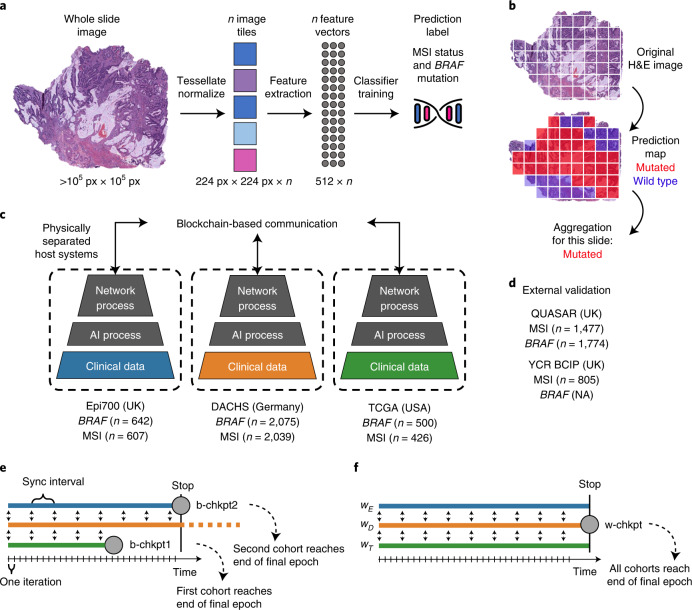
Schematic of the deep learning and SL workflows. **a**, Histology image analysis workflow for training. **b**, Histology image analysis workflow for model deployment (inference). **c**, SL workflow and training cohorts included in this study. On three physically separate bare-metal servers (dashed line), three different sets of clinical data reside. Each server runs an AI process (a program that trains a model on the data) and a network process (a program that handles communication with peers via blockchain). **d**, Test cohorts included in this study. **e**, Schematic of the basic SL experiment. For basic SL, the number of epochs is equal for all cohorts, and weights are equal for all cohorts. **f**, Schematic of the weighted SL experiment. For weighted SL, the number of epochs is larger for small cohorts, and weights are smaller for small cohorts (w_E_ = weight for the Epi700 cohort, w_D_ = weight for the DACHS cohort, w_T_ = weight for the TCGA cohort). Icon credits: **a**, OpenMoji (CC BY-SA 4.0); **c**,**d**, Twitter Twemoji (CC-BY 4.0).

**Extended Data Fig. 1:**
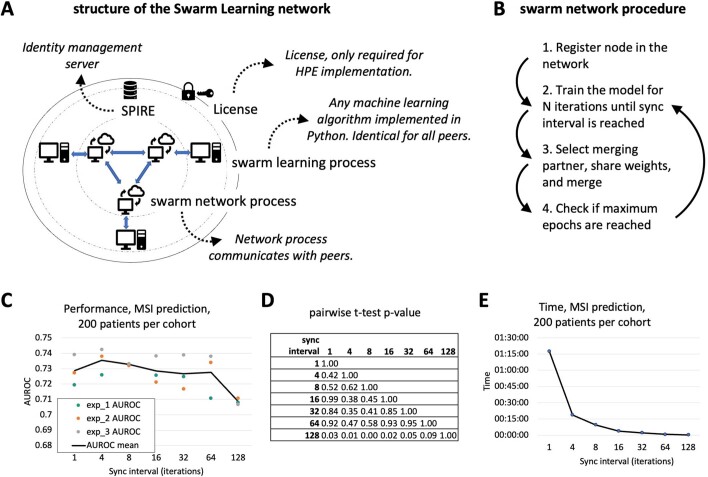
Workflow details and effect of synchronization interval. **(a)** Schematic of the structure of the Swarm Learning network in HPE Swarm Learning which was used in this study. **(b)** Schematic of the training procedure in Swarm Learning. **(c)** Evaluation of synchronization (sync) interval on the model performance. **(d)** Pairwise (two-sided) t-tests yielded non-significant (p > 0.05) p-values for all pairwise comparisons of the AUROCs obtained with 1, 4, 8, 16, 32 and 64 iterations between sync events. **(e)** Time for training with different sync internal. Abbreviations: WSI = whole slide images, MSI = microsatellite instability, SL = swarm learning, SN = swarm network, SPIRE = SPIFFE Runtime Environment. All statistical comparisons were made with two-sided t-tests without correction for multiple testing.

**Extended Data Fig. 2:**
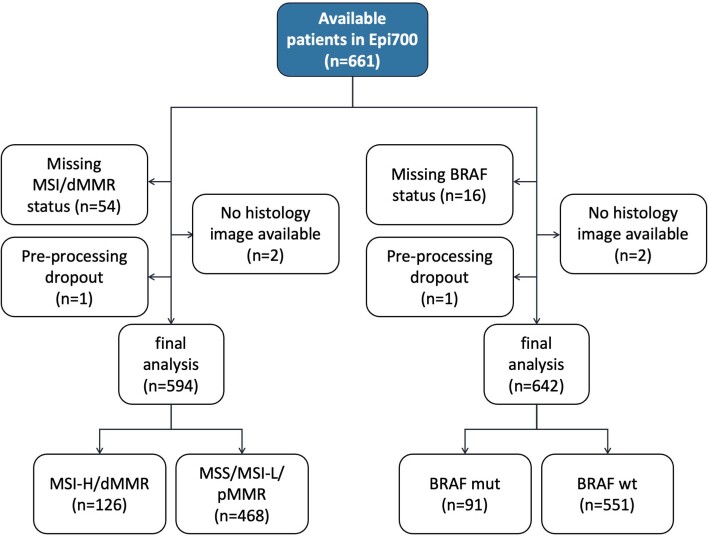
CONSORT chart for Epi700. Initial patient number in this dataset, exclusions and missing values, and final patient number.

**Extended Data Fig. 3:**
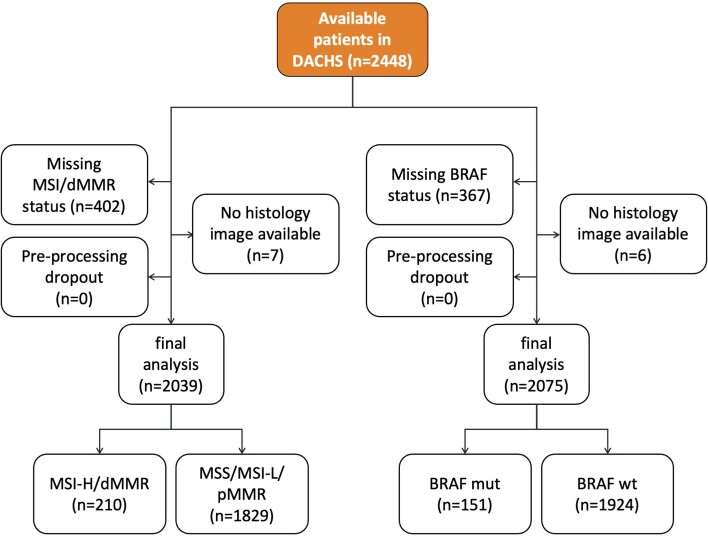
CONSORT chart for DACHS. Initial patient number in this dataset, exclusions and missing values, and final patient number.

**Extended Data Fig. 4:**
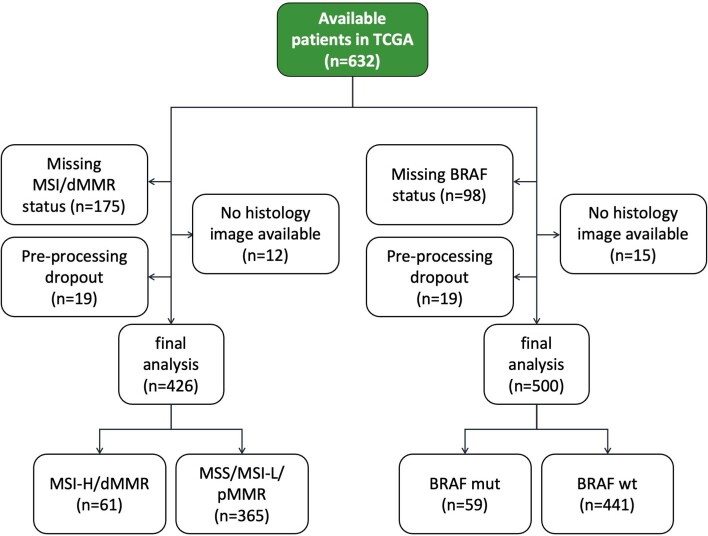
CONSORT chart for TCGA. Initial patient number in this dataset, exclusions and missing values, and final patient number.

We developed an SL-capable AI pipeline for molecular classification of solid tumors based on histopathology images (Fig. 1a,b and Extended Data Fig. 1a–e). We collected three large datasets for training: Epi700 (*n* = 661 patients from Northern Ireland; Extended Data Fig. 2), DACHS (Darmkrebs: Chancen der Verhütung durch Screening, *n* = 2,448 patients from southwestern Germany; Extended Data Fig. 3) and TCGA (The Cancer Genome Atlas, *n* = 632 patients; Fig. 1c, Table 1 and Extended Data Fig. 4). Each dataset was stored in a physically separate computing server. We then used our analysis pipeline in a retrospective multicenter study to predict genetic alterations directly from CRC histopathology whole slide images (WSIs), testing all models in external cohorts (Fig. 1d). First, we trained local AI models on each of the three training cohorts separately. Second, we compared their performances with that of a merged model, which was trained on all three training cohorts on a single computer. Third, we compared the performance of the merged model with the performance of three SL AI models. Basic model checkpoint 1 (b-chkpt1) was obtained when the partner with the smallest training cohort (TCGA) reached the end of the final epoch (Fig. 1e). Basic model checkpoint 2 (b-chkpt2) was obtained when the partner with the second-smallest training cohort (Epi700) reached the end of the final epoch. Finally, weighted SL balanced differences in cohort size by increasing the number of epochs for smaller cohorts while decreasing their weighting factor in the final model, yielding the weighted model checkpoint (w-chkpt) (Fig. 1f).

**Table 1:** Clinicopathological features of all cohorts

Variable	TCGA	DACHS	Epi700	YCR BCIP	QUASAR
Use in this study	Train	Train	Train	Test	Test
Cohort type	Population	Population	Population	Population	Clinical trial
No. of patients	632	2,448	661	889	2,190
Median age (years)	68	69	72.7	71	63
IQR for age (years)	18	14	14.5	15	12
Male	322 (50.9%)	1,436 (58.7%)	358 (54.2%)	494 (55.6%)	1,334 (60.9%)
Female	292 (46.2%)	1,012 (41.3%)	303 (45.8%)	395 (44.4%)	848 (38.7%)
Unknown sex	18 (2.85%)	0	0	0	8 (0.4%)
MSS/pMMR	392 (62%)	1,836 (75%)	471 (71.3%)	760 (85.5%)	1,529 (69.8%)
MSI/dMMR	65 (10.3%)	210 (8.6%)	136 (20.6%)	129 (14.5%)	246 (11.2%)
Unknown MSI status	175 (27.7%)	402 (16.4%)	54 (8.1%)	0	415 (19%)
MSI/MMR method	PCR 5-plex	PCR 3-plex	PCR 5-plex	IHC	IHC
Wild-type *BRAF*	471 (74.5%)	1,930 (78.8%)	553 (83.7%)	32 (3.6%^a^)	1,358 (62%)
Mutated *BRAF*	63 (10%)	151 (6.2%)	92 (13.9%)	75 (8.4%^a^)	129 (5.9%)
Unknown *BRAF* status	98 (15.5%)	367 (15%)	16 (2.4%)	782 (88%)	916 (41.8%)
*BRAF* detection method	Sequencing^37^	IHC, Sanger^38,39^	ColoCarta^[b,40^	NA	Pyrosequencing^41^
Stage I	76 (12%)	485 (19.8%)	0	169 (19%)	5 (0.2%)
Stage II	166 (26.3%)	801 (32.7%)	394 (59.6%)	317 (35.7%)	53 (2.4%)
Stage III	140 (22.2%)	822 (33.6%)	267 (40.4%)	370 (41.6%)	1,653 (75.5%)
Stage IV	63 (10%)	337 (13.8%)	0 (0%)	33 (3.7%)	268 (12.2%)
Stage unknown	187 (29.5)	3 (0.1%)	0	0	211 (9.7%)
Left-sided CRC	248 (39.2%)	1,607 (65.6%)	280 (42.3%)	487 (54.8%)	1,158 (52.9%)
Right-sided CRC	176 (27.8%)	819 (33.5%)	375 (56.7%)	332 (37.3%)	754 (34.4%)
Unknown side	209 (33%)	22 (0.9%)	6 (1%)	70 (7.9%)	278 (12.7%)

Right-sided CRC is defined as from cecum to transverse colon. IHC, immunohistochemistry; IQR, interquartile range; MMR, mismatch repair; NA, not available.

^a^*BRAF* testing in YCR BCIP was performed in only MSI/dMMR cases and was therefore not used as a prediction target in this study.

^b^The ColoCarta panel uses a validated mass spectrometry-based targeted screening panel of 32 somatic mutations in six genes (Agena Bioscience).

### SL models can predict *BRAF* mutational status

**Fig. 2:**
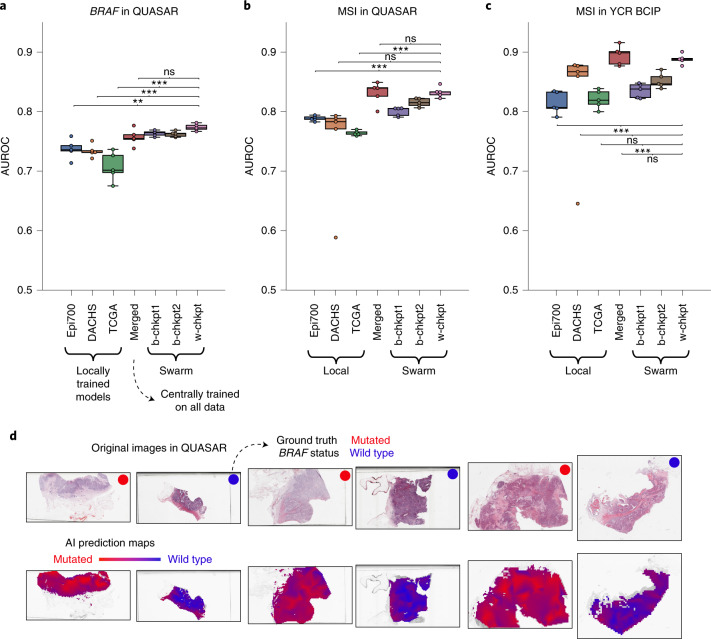
AI-based prediction of molecular alterations by local, merged and swarm models. **a**, Classification performance (AUROC) for prediction of *BRAF* mutational status at the patient level in the QUASAR dataset. Total cohort sizes (number of patients, for *BRAF* mutational status) in the training set are 642 for Epi700, 2,075 for DACHS and 500 for TCGA. Total cohort size (number of patients, for *BRAF* mutational status) in the test set is 1,477 for QUASAR. **b**, AUROC for prediction of MSI status in QUASAR. Total cohort sizes (number of patients, for MSI/dMMR) in the training sets are 594 for Epi700, 2,039 for DACHS and 426 for TCGA. Total cohort size (number of patients, for MSI/dMMR status) in the test set is 1,774 for QUASAR. **c**, AUROC for prediction of MSI status in the YCR BCIP dataset. Total cohort sizes (number of patients, for MSI/dMMR status) in the training sets are identical to those in **b**. Total cohort size (number of patients, for MSI/dMMR status) in the test set is 805 for YCR BCIP. In **a**–**c**, the boxes show the median values and quartiles, the whiskers show the rest of the distribution (except for points identified as outliers), and all original data points are shown. **d**, Model examination through slide heatmaps of tile-level predictions for representative cases in the QUASAR cohort. *P < 0.05; **P < 0.01; ***P < 0.001; ns, not significant (*P* > 0.05). Exact *P* values are available in Supplementary Table 1 (for **a**), Supplementary Table 2 (for **b**) and Supplementary Table 3 (for **c**). All statistical comparisons were made using two-sided *t*-tests without correction for multiple testing.

**Extended Data Fig. 5:**
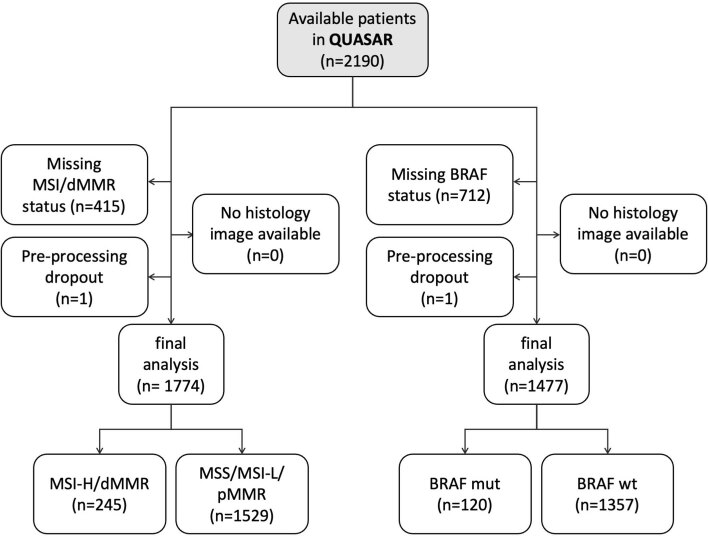
CONSORT chart for QUASAR. Initial patient number in this dataset, exclusions and missing values, and final patient number.

We evaluated the patient-level performance for prediction of *BRAF* mutational status on the QUASAR cohort (*n* = 1,774 patients from the United Kingdom; Extended Data Fig. 5). We found that local models achieved areas under the receiver operating curve (AUROCs; mean ± s.d.) of 0.7358 ± 0.0162, 0.7339 ± 0.0107 and 0.7071 ± 0.0243 when trained only on Epi700, DACHS and TCGA, respectively (Fig. 2a). Merging the three training cohorts on a central server (merged model) improved the prediction AUROC to 0.7567 ± 0.0139 (*P* = 0.0727 vs Epi700, *P* = 0.0198 vs DACHS, *P* = 0.0043 vs TCGA; Fig. 2a and Supplementary Table 1). This was compared with the performance of the SL AI models. b-chkpt1 achieved a prediction AUROC on the test set of 0.7634 ± 0.0047, which was significantly better than that of each local model (*P* = 0.0082 vs Epi700, *P* = 0.0005 vs DACHS, *P* = 0.0009 vs TCGA), but not significantly different from that of the merged model (*P* = 0.3433). b-chkpt2 achieved a similar performance: this model achieved an AUROC of 0.7621 ± 0.0045, which was significantly better than that of each local model (*P* = 0.0105 vs Epi700, *P* = 0.0006 vs DACHS, *P* = 0.0011 vs TCGA), and on par with that of the merged model (*P* = 0.4393). Finally, we assessed the performance of the weighted SL model (w-chkpt) for *BRAF* mutation prediction. In this task, w-chkpt achieved an AUROC of 0.7736 ± 0.0057. This is a significant improvement on the performances of all other models, including the local models of Epi700 (*P* = 0.0015), DACHS (*P* = 8.65 × 10^−5^) and TCGA (*P* = 0.0004), but also the merged model (*P* = 0.0374), b-chkpt1 (*P* = 0.0154) and b-chkpt2 (*P* = 0.0081; Supplementary Table 1).

### SL models can predict microsatellite instability

**Extended Data Fig. 6:**
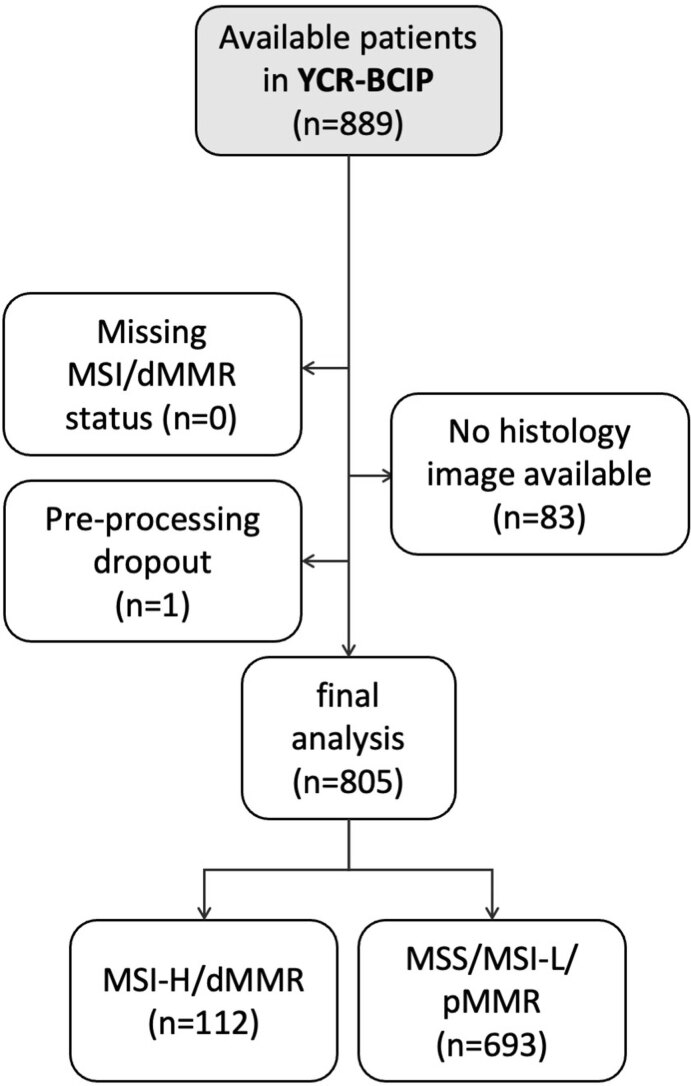
CONSORT chart for YCR-BCIP. Initial patient number in this dataset, exclusions and missing values, and final patient number.

Next, we tested our prediction pipeline in another benchmark task: the prediction of microsatellite instability (MSI)/mismatch repair deficiency (dMMR) status in the clinical trial cohort QUASAR (Fig. 2b) and the population-based cohort YCR BCIP (Yorkshire Cancer Research Bowel Cancer Improvement Programme; Fig. 2c and Extended Data Fig. 6). In QUASAR, b-chkpt1 and b-chkpt2 achieved prediction AUROCs of 0.8001 ± 0.0073 and 0.8151 ± 0.0071, respectively, and thereby significantly outperformed single-cohort models trained on Epi700 with an AUROC of 0.7884 ± 0.0043 (*P* = 0.0154 and *P* = 8.79 × 10^−5^ for b-chkpt1 and b-chkpt2, respectively; Supplementary Table 2). Similarly, SL outperformed MSI prediction models trained on TCGA with an AUROC of 0.7639 ± 0.0162 (*P* = 1.09 × 10^−5^ and *P* = 6.14 × 10^−7^ for b-chkpt1 and b-chkpt2, respectively). However, there was no significant difference between the model trained on the largest dataset (DACHS) and b-chkpt1 or b-chkpt2 in QUASAR (Fig. 2b) and YCR BCIP (Fig. 2c). For MSI prediction in QUASAR, w-chkpt significantly outperformed the local Epi700 model (*P* = 8.93 × 10^−6^) and the local TCGA model (*P* = 2.83 × 10^−7^), whereas the performance differences compared with the DACHS model were not statistically significant (DACHS AUROC = 0.8326 ± 0.0090 vs w-chkpt AUROC = 0.7403 ± 0.0878, *P* = 0.05705; Supplementary Table 2). Similar results were obtained for the second MSI validation dataset YCR BCIP (Supplementary Table 3). Compared with the merged model, w-chkpt was not significantly different for MSI prediction in QUASAR (merged AUROC = 0.8308 ± 0.0190 vs w-chkpt AUROC = 0.8326 ± 0.0089, *P* = 0.8650) or YCR BCIP (merged AUROC = 0.8943 ± 0.0161 vs w-chkpt AUROC = 0.8882 ± 0.0084, *P* = 0.4647). In other words, the performances of the merged model and w-chkpt were on par (Fig. 2b,c). Together, these data show that swarm-trained models consistently outperform local models and perform on par with centralized models in pathology image analysis.

### SL models are data efficient

**Fig. 3:**
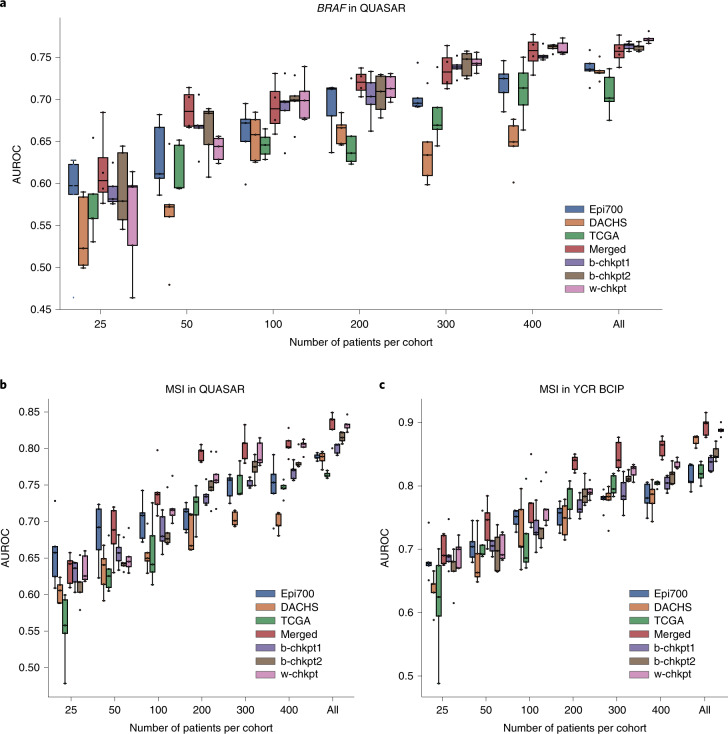
SL models are data efficient. **a**, Classification performance (AUROC) for prediction of *BRAF* mutational status at the patient level in the QUASAR cohort. Total cohort sizes (number of patients, for *BRAF* mutational status) in the training sets are 642 for Epi700, 2,075 for DACHS and 500 for TCGA. Total cohort size (number of patients, for *BRAF* mutational status) in the test set is 1,477 for QUASAR. **b**, Classification performance (AUROC) for prediction of MSI/dMMR status at the patient level in the QUASAR cohort. Total cohort sizes (number of patients, for MSI/dMMR) in the training sets are 594 for Epi700, 2,039 for DACHS and 426 for TCGA. Total cohort size (number of patients, for MSI/dMMR status) in the test set is 1,774 for QUASAR. **c**, Classification performance (AUROC) for prediction of MSI/dMMR status at the patient level in the YCR BCIP cohort. Total cohort sizes (number of patients, for MSI/dMMR status) in the training sets are identical to those in **b**. Total cohort size (number of patients, for MSI/dMMR status) in the test set is 805 for YCR BCIP. In **a**–**c**, the boxes show the median values and quartiles, the whiskers show the rest of the distribution (except for points identified as outliers), and all original data points are shown.

Learning from small datasets is a challenge in medical AI because prediction performance generally increases with increasing size of the training dataset^19,20^. Therefore, we investigated whether SL could compensate for the performance loss that occurs when only a small subset of patients from each institution is used for training. We found that restricting the number of patients in each training set to 400, 300, 200 and 100 markedly reduced prediction performance for single-dataset (local) models. For example, for prediction of *BRAF* mutational status in QUASAR, training on only a subset of patients in Epi700, DACHS or TCGA markedly reduced prediction performance and increased the model instability as evidenced by a larger interquartile range of predictions in experimental repetitions (Fig. 3a and Supplementary Table 4). In particular, for training *BRAF* prediction models on the largest cohort (DACHS), there was a pronounced performance drop from an AUROC of 0.7339 ± 0.0108 when training on all patients to an AUROC of 0.6626 ± 0.0162 when restricting the number of patients in the training set to 200. Performance losses for the model that was trained on the centrally merged data were less pronounced down to 50 patients per cohort (Fig. 3a). Strikingly, SL was also able to rescue the performance: down to 100 patients per cohort, weighted SL (w-chkpt) maintained a high performance with AUROCs of 0.7000 ± 0.0260 for 100 patients, 0.7139 ± 0.0149 for 200 patients and 0.7438 ± 0.0093 for 300 patients. The performances of these models were not statistically significantly different from that of the merged model (*P* = 0.7726, *P* = 0.7780, *P* = 0.2719 and *P* = 0.7130 for 100, 200, 300 and 400 patients, respectively; Fig. 3a). Similarly, b-chkpt1 and b-chkpt2 maintained high performance (comparable to that of the merged model) down to 100 patients per cohort. For MSI prediction in QUASAR, w-chkpt performance was comparable to that of the merged model down to 300 patients per cohort (*P* = 0.4342 and *P* = 0.7847 for 300 and 400 patients, respectively). For 200 patients or fewer, the merged model outperformed local models and swarm models (Fig. 3b and Supplementary Table 5). Similarly, for MSI prediction in YCR BCIP, single-cohort performance dropped as patients were dropped from the training set; the merged model and swarm models could partially rescue this performance loss, although the merged model outperformed the swarm models in this experiment (Fig. 3c and Supplementary Table 6). Together, these data show that SL models are highly resilient to small training datasets for prediction of *BRAF* mutational status, and partially resilient to small training datasets for prediction of MSI status.

### SL models learn plausible patterns

**Fig. 4:**
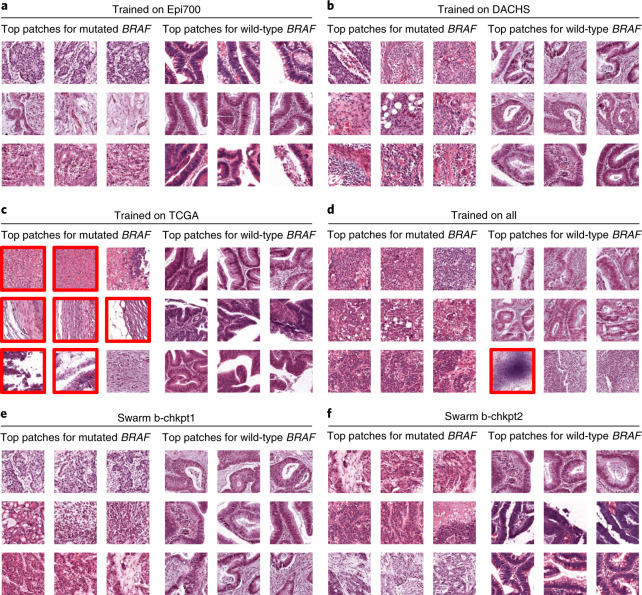
Highly predictive image patches for *BRAF* prediction. All patches are from the QUASAR test set and were obtained using the median model (out of five repetitions) trained on 300 randomly selected patients per training cohort. **a**–**f**, Model trained on Epi700 (**a**), model trained on DACHS (**b**), model trained on TCGA (**c**), model trained on all three datasets (**d**), swarm chkpt1 (**e**), swarm chkpt2 (**f**). Tiles with red borders contain artifacts or more than 50% nontumor tissue.

**Extended Data Fig. 7:**
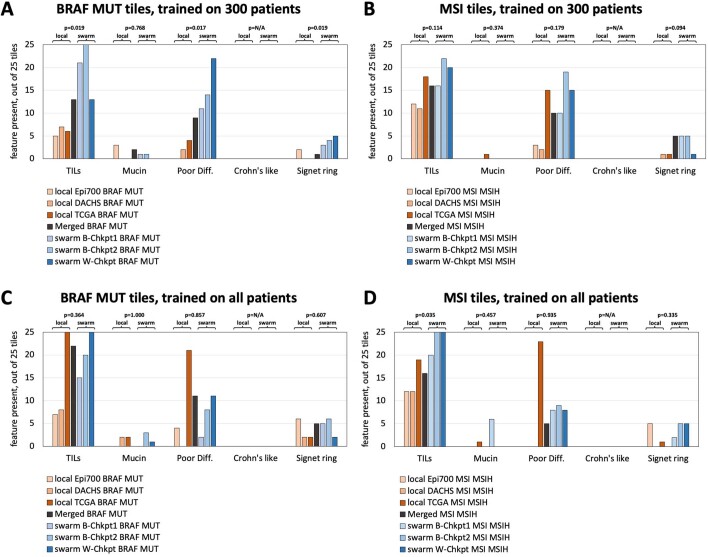
Results of the blinded reader study. For each model (seven model types, *BRAF* and MSI/dMMR prediction tasks, positive and negative class), a blinded observer scored the presence of five relevant histopathological patterns or structures in the highly scoring image tiles. **(a)** Presence of relevant patterns or structures in highly scoring tiles in the *BRAF* mutated class for *BRAF* prediction models trained on 300 patients per cohort, as scored by the blinded observer. P-values indicate a two-sided comparison between the three local models and the three Swarm-trained models for each feature. **(b)** Presence of relevant patterns or structures in highly scoring tiles in the *MSI/dMMR* for MSI status prediction models trained on 300 patients per cohort, as scored by the blinded observer. **(c)** Same experiment as panel (A), but for the models which were trained on all patients in all cohorts. **(d)** Same experiment as panel (B), but for the models which were trained on all patients in all cohorts. Abbreviations: MSI = mismatch repair deficiency, B-Chkpt = basic Swarm Learning experiment checkpoint, W-Chkpt = weighted Swarm Learning experiment checkpoint, TILs = tumor-infiltrating lymphocytes, Poor Diff. = poor differentiation, Crohn’s like = Crohn’s like lymphoid reaction, N/A = not applicable (division by zero). All statistical comparisons were made with two-sided t-tests without correction for multiple testing.

Medical AI models should not only have high performance, but should also be interpretable^28,29^. We assessed the model predictions on a millimeter scale by visualizing whole slide prediction heatmaps (Fig. 2d). These maps generally showed a clear and homogeneous predominance of one of the classes. In addition, we assessed the model predictions on a micrometer scale by extracting the image patches with the highest scores for models trained on 300 patients and all patients from the local training cohorts (Fig. 4a–c), the merged cohort (Fig. 4d) and the swarm models b-chkpt1, b-chkpt2 and w-chkpt (Fig. 4e,f). Qualitatively, we found that in many cases there was a histological phenotype known to be associated with either *BRAF* mutational status or MSI/dMMR, such as mucinous histology and/or poor differentiation^30,31^. However, we also observed that the highly scoring patches identified by the TCGA model failed to represent classical histopathological features of *BRAF* mutation; indeed, seven out of nine highly scoring tiles in this group showed abundant artifacts or no tumor tissue (Fig. 4c). The observation that such low-information patches were flagged by the model as being highly relevant shows that a model trained only on TCGA does not adequately learn to detect relevant patterns, possibly because of pronounced batch effects in the TCGA cohort^22^. We further investigated the plausibility of detected patterns through a systematic reader study, in which a blinded expert scored the presence of five relevant patterns or structures in 1,400 highly scoring image tiles: tumor-infiltrating lymphocytes (TILs), any mucus, poor differentiation, Crohn’s-like lymphoid reaction and signet ring cells. We found that out of all models trained on 300 patients per cohort, swarm-trained models frequently flagged image tiles with the presence of relevant patterns or structures, compared with locally trained models (Extended Data Fig. 7a,b). For *BRAF* prediction models, TILs (*P* = 0.019), poor differentiation (*P* = 0.017) and signet ring cells (*P* = 0.019) were significantly more frequently present in tiles selected by swarm-trained models than in those selected by locally trained models (Extended Data Fig. 7a). Similarly, for MSI/dMMR, these patterns were more abundant in tiles selected by swarm-trained models than in those selected by locally trained models, but these differences were not statistically significant (Extended Data Fig. 7b). For *BRAF* prediction models trained on all patients, we observed no significant difference in the abundance of relevant patterns or structures (Extended Data Fig. 7c). For MSI/dMMR prediction models trained on all patients, TILs were significantly (*P* = 0.035) more frequently present in tiles selected by swarm-trained models than in those selected by locally trained models (Extended Data Fig. 7d). In all image tiles for highly scoring tiles in the wild-type *BRAF* and microsatellite stability (MSS)/mismatch repair proficiency (pMMR) classes, the occurrence of relevant patterns or structures was uniformly low, and no statistically significant differences were present. Together, these data show that SL-based AI models can generate predictions that are explainable and plausible to human experts, and in some cases exceed the plausibility of locally trained models as assessed in a blinded study.

## Discussion

Currently, the total amount of healthcare data is increasing at an exponential pace. In histopathology, institutions across the world are digitizing their workflows, generating an abundance of data^6^. These image data can be used in new ways—for example, to make prognostic and predictive forecasts—with an aim to improve patient outcomes^3^. However, AI requires large and diverse datasets, and its performance scales with the amount of training data^19,20^. To train useful and generalizable AI models, institutions should be able to collaborate without jeopardizing patient privacy and information governance. In 2016, FL was proposed as a technical solution for such privacy-preserving distributed AI^32^. FL enables joint training of AI models by multiple partners who cannot share their data with each other. However, FL relies on a central coordinator who monopolizes the resulting AI model, concentrating the power of exploitation in the hands of a single entity. Thus, FL removes the need for data sharing but does not solve the problem of information governance. SL, however, offers a solution to the governance problem, providing a true collaborative and democratic approach in which partners communicate and work on the same level, jointly and equally training models and sharing the benefits^25,26,33^. Most recently, SL has been tested to detect coronavirus disease 2019 (COVID-19), tuberculosis, leukemia and lung pathologies from transcriptome analysis or X-ray images^26^. Here, we demonstrate that the use of SL can enable AI-based prediction of clinical biomarkers in solid tumors, and yields high-performing models for pathology-based prediction of *BRAF* and MSI status, two important prognostic and predictive biomarkers in CRC^3,9,34^. In the future, our approach could be applied to other image classification tasks in computational pathology. SL enables researchers to use small datasets to train AI models; co-training a model on many small datasets is equivalent to training a model on a single large dataset. This also reduces hardware requirements, potentially making SL an option for researchers in low-income and middle-income countries.

A possible technical limitation of our study is that we did not explicitly investigate differential privacy, but this could be incorporated in future work. Although histological images without their associated metadata are not considered protected health information even under the Health Insurance Portability and Accountability Act (HIPAA) in the United States^35^, any membership inference attack or model inversion attack from shared model weight updates can be precluded by implementing additional differential privacy measures^36^. Other technical improvements to the SL system are conceivable. For example, different weighting factors could be explored. A high-quality dataset could be weighted more than a low-quality dataset, and a more diverse dataset could be weighted more than a homogenous dataset. Another limitation of this work is that the model performance needs to be further improved before clinical implementation. Previous work has shown that when the sample size is increased to approximately 10,000 patients, classifier performance will increase^19,20^. Our study shows that SL enables multiple partners to jointly train models without sharing data, thereby potentially facilitating the collection of such large training cohorts. Finally, previous proof-of-concept studies on SL in medical AI relied on virtual machines on a single bare-metal device. Here, we improved this by using three physically separate devices and implementing our code largely with open-source software. Although this indicates that SL is feasible between physically distinct locations, embedding SL servers in existing healthcare infrastructure in different institutions in multiple countries would probably require substantial practical efforts, which should ideally be addressed in research consortia. To assess the interchangeability of model data generated by SL projects, validation of this technology in large-scale international collaborative efforts is needed. Our study provides a benchmark and a clear guideline for such future efforts, ultimately paving the way to establish SL in routine workflows.

## Methods

### Ethics statement

This study was carried out in accordance with the Declaration of Helsinki. This study is a retrospective analysis of digital images of anonymized archival tissue samples from five cohorts of patients with CRC. The collection and anonymization of patients in all cohorts took place in each contributing center. Ethical approval for research use of all cohorts was obtained from each contributing center. The MI-CLAIM (minimum information about clinical artificial intelligence modeling) checklist is available as Supplementary Table 7 (ref. ^29^).

### Patient cohorts

We collected digital WSIs of H&E-stained slides of archival tissue sections of human CRC from five patient cohorts, three of which were used as training cohorts and two of which were used as test cohorts (Table 1). The value proposition of SL is to enable geographically distributed partners to co-train models without data exchange. Hence, we selected three geographically distributed training cohorts, representative of various real-world clinical settings: (1) the Northern Ireland Epi700 cohort (*n* = 661; Extended Data Fig. 2) of patients with stage II and stage III colon cancer, whose data were provided by the Northern Ireland Biobank^40,42^ (application NIB20-0346); (2) the DACHS cohort (*n* = 2,448; Extended Data Fig. 3), including samples from patients with CRC at any disease stage recruited at more than 20 hospitals in Germany for a large population-based case-control study, which is coordinated by the German Cancer Research Center (DKFZ)^43–45^; and (3) the TCGA CRC cohort (*n* = 632; Extended Data Fig. 4), a large collection of tissue specimens from several populations in study centers across different countries, but largely from the United States (https://portal.gdc.cancer.gov). The first test cohort was derived from a clinical trial of adjuvant therapy, the QUASAR trial (*n* = 2,206, Extended Data Fig. 5), which originally aimed to determine the survival benefit from adjuvant chemotherapy in patients with CRC from the United Kingdom^41,46^. The second test cohort was the YCR BCIP^47^ cohort (*n* = 889 surgical resection slides; Extended Data Fig. 6), from a population-based study collected in Yorkshire in the United Kingdom. For all cohorts, *BRAF* mutational status and MSI/dMMR^48^ data were acquired. Despite the different geographic origins, the distribution of tumor stages in TCGA, DACHS and YCR BCIP is similar (Table 1), whereas in QUASAR, stage III tumors are overrepresented, as adjuvant therapy is mainly indicated in stage III tumors. We deliberately selected YCR BCIP and QUASAR as test cohorts to investigate the robustness of the AI models both on a general population and on a clinical trial population; in a clinical trial population, determining molecular status is highly relevant for evaluation of treatment efficacy. As the ground truth diagnostic methods for MSI/dMMR, immunohistochemistry was used in YCR BCIP and QUASAR, and PCR was used in TCGA, DACHS (ref. ^49^) and Epi700 (ref. ^40^). As the ground truth diagnostic methods for *BRAF* mutational status, immunohistochemistry and Sanger sequencing were used in DACHS (refs. ^38,39^), and pyrosequencing was used in QUASAR. In Epi700, *BRAF* mutation screening was performed as part of the ColoCarta panel using a validated mass spectrometry-based targeted screening panel of 32 somatic mutations in six genes (Agena Bioscience)^40^. These ground truth diagnostic methods are the clinical state of the art in determining MSI/dMMR status^50^. In YCR BCIP, analysis of *BRAF* was only undertaken for dMMR tumors, and *BRAF* mutational status was therefore not assessed in this cohort in the current study. A CONSORT (Consolidated Standards of Reporting Trials) flowchart for each cohort is available as Extended Data Figs. 2–7 (ref. ^51^). There was no overlap between the training cohorts and test cohorts.

### Principle of SL

The principle of SL is to jointly train a machine learning model in different physically separated computer systems. Here, we use SL in a network of three physically separate computers (peers). Model weights are sent from each partner to the other peers at multiple synchronization (sync) events, which happen at the end of each sync interval. Model weights are averaged at each sync event, before the training continues at each peer with the averaged parameters. Unlike in FL, there is no central instance that always merges the parameters. Instead, smart contracts on an Ethereum blockchain (https://ethereum.org) enable the network to select any of the peers to perform parameter merging at every sync stop. In this setup, the blockchain maintains the global state information about the model. We designed, applied and evaluated two types of SL: basic and weighted. Basic SL is a simple procedure; assume that the training datasets A, B and C each have a different number of patients (A < B < C). We train on all datasets for the same fixed number of epochs (five epochs, motivated by previous studies). The system holding dataset A will reach the final epoch faster than those holding datasets B and C. At this point, the basic model checkpoint b-chkpt1 is created. The systems holding datasets B and C will continue until B reaches the final epoch. At this point, the basic model checkpoint b-chkpt2 is created. Also at this point, the system holding dataset C will stop, because at least two partners are required by default. However, the fact that all three systems reach the final epoch at different time points may be suboptimal; it would make sense to train all datasets for the same time, until they all stop at the same point in time. We have done this and termed it ‘weighted SL’, generating w-chkpt. This implies that smaller datasets will be passed through the network more times than larger datasets. To compensate for this, smaller datasets receive a lower weighting factor. The weighting factor is strictly proportional to the number of tiles.

### SL implementation

Here, we use the Hewlett Packard Enterprise (HPE) implementation of Swarm Learning (‘master’ release of 10 June 2021), which has four components: the SL process, the swarm network process, identity management and HPE license management^26^. All processes (also called “nodes” in the original HPE implementation) run in a Docker container. The key component is the SL process, which contains the image processing components (Extended Data Fig. 1a). The SL process sends the model weights to the swarm network process. The swarm network process handles peer crosstalk over the network. For identity management, we used SPIRE (Secure Production Identity Framework for Everyone (SPIFFE) Runtime Environment). A detailed hands-on description of this process with a small example dataset and step-by-step instructions to reproduce our experiments is available at https://github.com/KatherLab/SWARM (instructions for troubleshooting, and a mechanism for users to report issues are also available). Our SL setup can also be executed on a cluster with tasks potentially queued. The participating peers coordinate the synchronization among each other such that the other peers will wait if one peer is not yet ready for synchronization. However, as this might be inefficient in terms of computational resources (the other peers are idle if the task of one peer is queued), we recommend executing our SL setup on dedicated computers, or giving high priority to the execution when performed on clusters.

### Image preprocessing and deep learning

For prediction of molecular features from image data, we adapted our weakly supervised end-to-end prediction pipeline, which outperformed similar approaches for mutation prediction in a recent benchmark study^52^. As an implementation of this pipeline, we used our own image processing library, Histopathology Image Analysis (HIA)^9^. Histopathological WSIs were acquired in SVS format. As a preprocessing step, high-resolution WSIs were tessellated into patches of 512 pixels × 512 pixels × 3 colors and were color-normalized^53^. During this process, blurry patches and patches with no tissue are removed from the dataset using Canny edge detection^52^. Specifically, we obtained a normalized edge image using the Canny() method in Python’s OpenCV package (version 4.1.2) and then removed all tiles with a mean value below a threshold of 4. Subsequently, we used ResNet-18 to extract a 512 × 1 feature vector from 150 randomly selected patches for each patient, as previous work showed that 150 patches are sufficient to obtain robust predictions^9^. Before training, the number of tiles in each class was equalized by random undersampling, as described before^9,12^. Feature vectors and patient-wise target labels (*BRAF* or MSI status) served as input to a fully connected classification network. The classification network comprised four layers with 512 × 256, 256 × 256, 256 × 128 and 128 × 2 connections with a rectified linear unit (ReLU) activation function. This approach is a re-implementation of a previously published workflow^52^. Only one model was developed and used, and no other models were evaluated. Only one set of hyperparameters was used (Supplementary Table 8) to train the deep learning model (based on a previous study^52^).

### Optimizing efficiency of model synchronization

Different choices of sync intervals were evaluated on the QUASAR MSI/dMMR prediction task, but not on any of the other prediction tasks. This was evaluated for a single model, a simple swarm model trained on 200 random patients from each training cohort, repeated three times with different random seeds. The sync interval did not have a significant effect on classification performance in the range of 1 to 64 iterations between sync events (Extended Data Fig. 1c,d). The training time decreased with more frequent synchronizations (Extended Data Fig. 1e), indicating that the SL time was dominated by network communication overhead (Extended Data Fig. 1e). For all further experiments, we used a sync interval of four iterations.

### Experimental design and statistics

First, we trained MSI and *BRAF* classifiers on each of the training cohorts individually. Second, all training cohorts were merged, and new classifiers were trained on the merged cohort (combining all three training cohorts in a single computer system). Third, classifiers were trained by SL, with the SL training process initiated on three separate bare-metal servers containing one training cohort each. Fourth, all models were externally validated on the validation cohorts. Two variants of SL were explored (baseline SL and weighted SL), as explained above. For baseline SL, each cohort was trained for a fixed number of epochs, and two resulting models were saved at two checkpoints (b-chkpt1 and b-chkpt2). b-chkpt1 was reached when the smallest cohort concluded the final epoch, and b-chkpt2 was reached when the second-smallest cohort concluded the final epoch. In weighted SL, only one model checkpoint is generated (w-chkpt). Finally, to investigate data efficiency, we repeated all experiments for subsets of 25, 50, 100, 200, 300 and 400 patients per cohort, randomly selected in a stratified way (preserving class proportions). All experiments were repeated five times with different random seeds. AUROC was selected as the primary metric to evaluate algorithm performance and potential clinical utility. AUROC is the most widely used evaluation criterion for binary classification tasks in computational pathology and was chosen to enable a comparison with the findings of previous studies^54^. The AUROCs of five training runs (technical replicates with different random seeds) of a given model were compared. A two-sided unpaired *t*-test with *P* ≤ 0.05 was considered statistically significant. The raw results of all experimental repetitions are available in Supplementary Data 1.

### Model examination techniques

To examine the plausibility of model predictions^29^, we used three methods: whole slide prediction heatmaps; a qualitative analysis of highly scoring image tiles (patches); and a quantitative, blinded, reader study of highly scoring image tiles. First, whole slide prediction heatmaps were generated by visualizing the model prediction as a continuous value with a univariate color map, linearly interpolating gaps. For whole slide prediction heatmaps, the models with median performance (from five models) for all model types (three local, one merged and three swarm) trained on all patients were used. Second, highly scoring image tiles were generated by using the *N* highest-scoring tiles from the *M* highest-scoring patients as described before^12^ and were qualitatively checked for plausibility. Qualitative plausibility criteria were as follows: (1) Is tumor present on the highly scoring tiles?; (2) Are highly scoring tiles free of artifacts?; and (3) Is the phenotype subjectively consistent with a histological phenotype associated with *BRAF* mutations and/or MSI/dMMR? These criteria were assessed in highly scoring image tiles generated by the median model (median performance out of five replicates) for each model type (three local, one merged and three swarm), using the model that was trained on all patients, as well as the model that was trained on 300 patients per cohort. Third, highly scoring image tiles were systematically evaluated by an expert observer (S.F.) in a blinded study. In this study, the five highest-scoring tiles for the five highest-scoring patients for mutated and wild-type *BRAF* and MSI/dMMR and MSS/pMMR (1,400 image tiles total) were assessed for the presence of TILs, the presence of any mucin, poor differentiation, Crohn’s-like lymphoid reaction and the presence of signet ring cells, based on criteria proposed in ref. ^31^. Again, for the qualitative reader study, the model with the median performance out of five replicates was used.

### Hardware

In our setup, three computer systems (all consumer hardware) were used for the SL experiments. In detail, the systems had the following specifications: system A, 128 GB RAM and two NVIDIA Quadro RTX 6000 graphics processing units (GPUs); system B, 64 GB RAM and one NVIDIA RTX A6000 GPU; and system C, 64 GB RAM and two NVIDIA Quadro RTX 6000 GPUs. All of the systems accessed a 1 GBit s^−1^ Internet connection.

### Reporting Summary

Further information on research design is available in the Nature Research Reporting Summary linked to this article.

## Online content

Any methods, additional references, Nature Research reporting summaries, source data, extended data, supplementary information, acknowledgements, peer review information; details of author contributions and competing interests; and statements of data and code availability are available at 10.1038/s41591-022-01768-5.

## Supplementary information

Supplementary Information Supplementary Tables S1–S8.

Reporting Summary

Supplementary Data 1 Performance results of all experiments related to Fig. 2 and Fig. 4. All statistical comparisons were made with two-sided *t*-tests without correction for multiple testing.

Supplementary Data 2 Raw data for Extended Data Fig. 1.

Supplementary Data 3 Raw data for Extended Data Fig. 2.

## Data Availability

Some of the data that support the findings of this study are publicly available, and some are proprietary datasets provided under collaboration agreements. All data (including histological images) from the TCGA database are available at https://portal.gdc.cancer.gov. All molecular data for patients in the TCGA cohorts are available at https://cbioportal.org. Data access for the Northern Ireland Biobank can be requested at http://www.nibiobank.org/for-researchers. All other data are under controlled access according to the local ethical guidelines and can only be requested directly from the respective study groups that independently manage data access for their study cohorts. Access to QUASAR and YCR BCIP was obtained via Pathology & Data Analytics, Leeds Institute of Medical Research at St James’s, University of Leeds, Leeds, UK (https://medicinehealth.leeds.ac.uk/dir-record/research-groups/557/pathology-and-data-analytics), and access to DACHS was obtained via the DACHS study group at http://dachs.dkfz.org/dachs/kontakt.html.

## Code availability

Our source codes are available with an example dataset, detailed instructions and troubleshooting help at https://github.com/KatherLab/SWARM. All source codes for the baseline HIA workflow are available at https://github.com/KatherLab/HIA. All source codes for image preprocessing are available at https://github.com/KatherLab/preProcessing. Our SL implementation requires HPE’s SL community edition, which is publicly available under an Apache 2.0 license along with detailed instructions and troubleshooting help at https://github.com/HewlettPackard/swarm-learning.
