# Supplementary material for: Swarm learning for decentralized artificial intelligence in cancer histopathology
Source: Nat Med. 2022 Apr 25;28(6):1232–9. doi: 10.1038/s41591-022-01768-5 (PMC9205774; doi:10.1038/s41591-022-01768-5)
Supplement: Supplementary file 1 — Supplementary Tables S1–S8. [file 41591_2022_1768_MOESM1_ESM.pdf]

nature portfolio

https://doi.org/10.1038/s41591-022-01768-5

Supplementary information

Swarm learning for decentralized artificial intelligence in cancer histopathology

In the format provided by the authors and unedited

	Epi700	DACHS	TCGA	Merged	b-chkpt1	b-chkpt2	w-chkpt
**Epi700**	1	0.735447	0.054133	0.072777	0.008285	0.010536	0.001469
**DACHS**	0.735447	1	0.056813	0.019865	0.000511	0.000663	8.65E-05
**TCGA**	0.054133	0.056813	1	0.004342	0.000997	0.001145	0.000358
**Merged**	0.072777	0.019865	0.004342	1	0.343359	0.439399	0.037475
**b-chkpt1**	0.008285	0.000511	0.000997	0.343359	1	0.673565	0.015439
**b-chkpt2**	0.010536	0.000663	0.001145	0.439399	0.673565	1	0.00812
**w-chkpt**	0.001469	8.65E-05	0.000358	0.037475	0.015439	0.00812	1

Supplementary Table S1: P-values for pairwise comparisons between experiments for BRAF prediction in QUASAR for all patients (unpaired, two-sided t-test).

	Epi700	DACHS	TCGA	Merged	b-chkpt1	b-chkpt2	w-chkpt
**Epi700**	1	0.300247	1.54E-05	0.001257	0.015433	8.79E-05	8.93E-06
**DACHS**	0.300247	1	0.64237	0.064677	0.199045	0.112427	0.057049
**TCGA**	1.54E-05	0.64237	1	5.80E-05	1.09E-05	6.14E-07	2.83E-07
**Merged**	0.001257	0.064677	5.80E-05	1	0.009638	0.120348	0.864977
**b-chkpt1**	0.015433	0.199045	1.09E-05	0.009638	1	0.010362	0.000234
**b-chkpt2**	8.79E-05	0.112427	6.14E-07	0.120348	0.010362	1	0.008928
**w-chkpt**	8.93E-06	0.057049	2.83E-07	0.864977	0.000234	0.008928	1

Supplementary Table S2: P-values for pairwise comparisons between experiments for MSI prediction in QUASAR for all patients (unpaired, two-sided t-test).

	Epi700	DACHS	TCGA	Merged	b-chkpt1	b-chkpt2	w-chkpt
**Epi700**	1	0.810466	0.562843	8.88E-05	0.06603	0.005866	4.33E-05
**DACHS**	0.810466	1	0.919589	0.169501	0.833261	0.575862	0.203239
**TCGA**	0.562843	0.919589	1	8.08E-05	0.135212	0.008523	2.87E-05
**Merged**	8.88E-05	0.169501	8.08E-05	1	0.000171	0.001703	0.464731
**b-chkpt1**	0.06603	0.833261	0.135212	0.000171	1	0.065841	4.10E-05
**b-chkpt2**	0.005866	0.575862	0.008523	0.001703	0.065841	1	0.000734
**w-chkpt**	4.33E-05	0.203239	2.87E-05	0.464731	4.10E-05	0.000734	1

Supplementary Table S3: P-values for pairwise comparisons between experiments for dMMR prediction in YCR-BCIP for all patients (unpaired, two-sided t-test).

	Epi700	DACHS	TCGA	Merged	b-chkpt1	b-chkpt2	w-chkpt
**Epi700**	1	0.124088	0.15321	0.164606	0.578819	0.42984	0.208304
**DACHS**	0.124088	1	0.655429	0.000639	0.02226	0.008388	0.000813
**TCGA**	0.15321	0.655429	1	0.013389	0.059135	0.038283	0.016248
**Merged**	0.164606	0.000639	0.013389	1	0.339588	0.438796	0.778013
**b-chkpt1**	0.578819	0.02226	0.059135	0.339588	1	0.807427	0.435173
**b-chkpt2**	0.42984	0.008388	0.038283	0.438796	0.807427	1	0.567631
**w-chkpt**	0.208304	0.000813	0.016248	0.778013	0.435173	0.567631	1

Supplementary Table S4: P-values for pairwise comparisons between experiments for BRAF prediction in QUASAR for 200 patients (unpaired, two-sided t-test).

	Epi700	DACHS	TCGA	Merged	b-chkpt1	b-chkpt2	w-chkpt
**Epi700**	1	0.113646	0.381066	1.44E-05	0.021346	0.01977	0.008786
**DACHS**	0.113646	1	0.05067	3.55E-05	0.004533	0.004592	0.002457
**TCGA**	0.381066	0.05067	1	0.000443	0.274513	0.114665	0.059786
**Merged**	1.44E-05	3.55E-05	0.000443	1	7.55E-05	0.017031	0.029182
**b-chkpt1**	0.021346	0.004533	0.274513	7.55E-05	1	0.314582	0.152971
**b-chkpt2**	0.01977	0.004592	0.114665	0.017031	0.314582	1	0.730893
**w-chkpt**	0.008786	0.002457	0.059786	0.029182	0.152971	0.730893	1

Supplementary Table S5: P-values for pairwise comparisons between experiments for MSI prediction in QUASAR for 200 patients (unpaired, two-sided t-test).

	**Epi700**	**DACHS**	**TCGA**	**Merged**	**b-chkpt1**	**b-chkpt2**	**w-chkpt**
**Epi700**	1	0.605611	0.072281	4.92E-05	0.229145	0.022538	0.005563
**DACHS**	0.605611	1	0.06765	0.000356	0.175667	0.0306	0.01444
**TCGA**	0.072281	0.06765	1	0.00249	0.302962	0.679152	0.458176
**Merged**	4.92E-05	0.000356	0.00249	1	6.96E-05	0.001888	0.00042
**b-chkpt1**	0.229145	0.175667	0.302962	6.96E-05	1	0.108632	0.026695
**b-chkpt2**	0.022538	0.0306	0.679152	0.001888	0.108632	1	0.746109
**w-chkpt**	0.005563	0.01444	0.458176	0.00042	0.026695	0.746109	1

Supplementary Table S6: P-values for pairwise comparisons between experiments for dMMR prediction in YCR-BCIP for 200 patients (unpaired, two-sided t-test).

Study design (Part 1)	Completed, page	
The clinical problem in which the model will be employed is clearly detailed in the paper.	yes	3
The research question is clearly stated.	yes	3
The characteristics of the cohorts (training and test sets) are detailed in the text.	yes	8
The cohorts (training and test sets) are shown to be representative of real-world clinical settings.	yes	8
The state-of-the-art solution used as a baseline for comparison has been identified and detailed.	yes	8
**Data and optimization (Parts 2, 3)**		
The origin of the data is described and the original format is detailed in the paper.	yes	10
Transformations of the data before it is applied to the proposed model are described.	yes	10
The independence between training and test sets has been proven in the paper.	yes	8
Details on the models that were evaluated and the code developed to select the best model are provided.	yes	9
Is the input data type structured or unstructured?: **Unstructured images**		
**Model performance (Part 4)**		
The primary metric selected to evaluate algorithm performance (e.g., AUC, F-score, etc.), including the justification for selection, has been clearly stated.	yes	10
The primary metric selected to evaluate the clinical utility of the model (e.g., PPV, NNT, etc.), including the justification for selection, has been clearly stated.	yes	10
The performance comparison between baseline and proposed model is presented with the appropriate statistical significance.	yes	4
**Model examination (Part 5)**		
Examination technique 1a: **Whole slide prediction heatmaps**	yes	10
Examination technique 2a: **Highly scoring tiles (qualitative and quantitative analysis)**	yes	10

A discussion of the relevance of the examination results with respect to model/algorithm performance is presented.	yes	6
A discussion of the feasibility and significance of model interpretability at the case level if examination methods are uninterpretable is presented.	yes	6
A discussion of the reliability and robustness of the model as the underlying data distribution shifts is included.	yes	4
**Reproducibility (Part 6)**		
Tier 1: complete sharing of the code (https://github.com/KatherLab/SWARM)		

Supplementary Table S7: Minimum information about clinical artificial intelligence modeling (MI-CLAIM) checklist.

**Category**	**Hyperparameter**	**Value**	**Reference**
HIA	Learning Rate	0.0001	Ghaffari Laleh et al.[53]
HIA	Batch size	124	
HIA	Number of Epochs	5	
HIA	Optimizer	Adam	
HIA	Network model	ResNet18	
HIA	Tiles per slide	150	Kather et al.[9]
Swarm	Sync interval	4	hyperparameter optimization
Swarm	min peers	2	https://github.com/HewlettPackard/swarm-learning
Swarm	adaptive sync	false	
Swarm	node weights	1	

Supplementary Table S8: HIA and swarm learning hyperparameters.
