# Supplementary material for: Swarm learning for decentralized artificial intelligence in cancer histopathology
Source: Nat Med. 2022 Apr 25;28(6):1232–9. doi: 10.1038/s41591-022-01768-5 (PMC9205774; doi:10.1038/s41591-022-01768-5)
Supplement: Supplementary file 2 — Reporting Summary [file 41591_2022_1768_MOESM2_ESM.pdf]

nature portfolio

Corresponding author(s): Jakob Nikolas Kather

Last updated by author(s): Feb 18, 2022

Reporting Summary

Nature Portfolio wishes to improve the reproducibility of the work that we publish. This form provides structure for consistency and transparency in reporting. For further information on Nature Portfolio policies, see our Editorial Policies and the Editorial Policy Checklist.

Statistics

For all statistical analyses, confirm that the following items are present in the figure legend, table legend, main text, or Methods section.

n/a	Confirmed	
☐	☒	The exact sample size (*n*) for each experimental group/condition, given as a discrete number and unit of measurement
☐	☒	A statement on whether measurements were taken from distinct samples or whether the same sample was measured repeatedly
☐	☒	The statistical test(s) used AND whether they are one- or two-sided<br>*Only common tests should be described solely by name; describe more complex techniques in the Methods section.*
☒	☐	A description of all covariates tested
☐	☒	A description of any assumptions or corrections, such as tests of normality and adjustment for multiple comparisons
☐	☒	A full description of the statistical parameters including central tendency (e.g. means) or other basic estimates (e.g. regression coefficient) AND variation (e.g. standard deviation) or associated estimates of uncertainty (e.g. confidence intervals)
☐	☒	For null hypothesis testing, the test statistic (e.g. *F*, *t*, *r*) with confidence intervals, effect sizes, degrees of freedom and *P* value noted<br>*Give P values as exact values whenever suitable.*
☒	☐	For Bayesian analysis, information on the choice of priors and Markov chain Monte Carlo settings
☐	☒	For hierarchical and complex designs, identification of the appropriate level for tests and full reporting of outcomes
☒	☐	Estimates of effect sizes (e.g. Cohen's *d*, Pearson's *r*), indicating how they were calculated

Our web collection on statistics for biologists contains articles on many of the points above.

Software and code

Policy information about availability of computer code

Data collection: No specific software was used to collect data.

Data analysis: Our source codes are available with an example dataset and detailed instructions and troubleshooting at https://github.com/KatherLab/SWARM. All source codes for the baseline histology image analysis (HIA) workflow are available at https://github.com/KatherLab/HIA. All source codes for image preprocessing are available at https://github.com/KatherLab/preProcessing. Our SL implementation requires the "SL community edition" by Hewlett Packard Enterprise (Spring, Texas, United States), which is publicly available under an Apache 2.0 license along with detailed instructions and troubleshooting at https://github.com/HewlettPackard/swarm-learning.

For manuscripts utilizing custom algorithms or software that are central to the research but not yet described in published literature, software must be made available to editors and reviewers. We strongly encourage code deposition in a community repository (e.g. GitHub). See the Nature Portfolio guidelines for submitting code & software for further information.

Data

Policy information about availability of data

All manuscripts must include a data availability statement. This statement should provide the following information, where applicable:

- Accession codes, unique identifiers, or web links for publicly available datasets
- A description of any restrictions on data availability
- For clinical datasets or third party data, please ensure that the statement adheres to our policy

The data that support the findings of this study are in part publicly available, in part proprietary da-tasets provided under collaboration agreements. All data (including histological images) from the TCGA database are available at https://portal.gdc.cancer.gov/. All molecular data for patients in the TCGA cohorts are available at https://cbioportal.org. Data access for the Northern Ireland Biobank can be requested at http://www.nibiobank.org/for-researchers. All other data are

under controlled ac-cess according to the local ethical guidelines and can only be requested from the respective study groups directly who independently manage data access for their study cohorts. Access to QUASAR and YCR-BCIP was obtained via “Pathology & Data Analytics, Leeds Institute of Medical Research at St James's, University of Leeds, Leeds, United Kingdom” (https://medicinehealth.leeds.ac.uk/dir-record/research-groups/557/pathology-and-data-analytics) and access to DACHS was obtained via the DACHS study group at http://dachs.dkfz.org/dachs/kontakt.html.

Field-specific reporting

Please select the one below that is the best fit for your research. If you are not sure, read the appropriate sections before making your selection.

- [x] Life sciences
- [ ] Behavioural & social sciences
- [ ] Ecological, evolutionary & environmental sciences

For a reference copy of the document with all sections, see nature.com/documents/nr-reporting-summary-flat.pdf

Life sciences study design

All studies must disclose on these points even when the disclosure is negative.

Sample size	Three training cohorts Epi700 (n=661 patients), DACHS (n=2448) and TCGA (n=632). Two validation cohorts QUASAR (n=2206) and YCR-BCIP (n=889). No specific procedure to determine the sample size was employed. These sample sizes were deemed sufficient because they are larger than or in the same range as most similar studies in the scientific literature, as summarized by Echle et al. (https://doi.org/10.1016/j.immuno.2021.100008).
Data exclusions	Reasons for data exclusion were missing image data, missing molecular data and faulty image files resulting in pre-processing dropout. All dropouts are listed in Suppl. Figures S2-S6.
Replication	We repeated all experiments five times with different random seeds. All attempts at replication were successful.
Randomization	Random seeds were generated with Python's random number generator. Samples were randomly allocated to any experimental groups.
Blinding	As part of our study, we performed a reader study in which an expert observer evaluated image tiles. This observer was blinded during this evaluation. No other parts of our study required blinding because no subjective evaluation by an observer was involved in these parts of our study.

Reporting for specific materials, systems and methods

We require information from authors about some types of materials, experimental systems and methods used in many studies. Here, indicate whether each material, system or method listed is relevant to your study. If you are not sure if a list item applies to your research, read the appropriate section before selecting a response.

Materials & experimental systems

n/a	Involved in the study	
[x]	[ ]	Antibodies
[x]	[ ]	Eukaryotic cell lines
[x]	[ ]	Palaeontology and archaeology
[x]	[ ]	Animals and other organisms
[x]	[ ]	Human research participants
[ ]	[x]	Clinical data
[x]	[ ]	Dual use research of concern

Methods

n/a	Involved in the study	
[x]	[ ]	ChIP-seq
[x]	[ ]	Flow cytometry
[x]	[ ]	MRI-based neuroimaging

Clinical data

Policy information about clinical studies

All manuscripts should comply with the ICMJE guidelines for publication of clinical research and a completed CONSORT checklist must be included with all submissions.

Clinical trial registration	No clinical trial was performed. In this study, we retrospectively used consecutive data from the routine clinical database.
Study protocol	No formal study protocol is available. The main analysis steps follow a procedure which has been previously established by Kather et al., Nature Medicine, 2019 (DOI 10.1038/s41591-019-0462-y) and Warnat-Herresthal et al., Nature, 2021 (DOI 10.1038/s41586-021-03583-3).
Data collection	We collected digital whole slide images (WSI) of H&E-stained slides of archival tissue sections of human colorectal cancer (CRC) from five patient cohorts (clinico-pathological characteristics in Table 1).
Outcomes	The primary endpoint for this study was the area under the receiver operator characteristic curve (AUROC) for detection of binary categorical outputs. The AUROCs of five training runs of a given model were compared. A two-tailed unpaired t-test with p<= 0.05

was considered statistically significant. In the manuscript, AUROCs are given as mean +/- standard deviation. All raw results of all experimental repetitions are available in Suppl. Table S9.
